# Association between *Helicobacter pylori* Infection and Nonalcoholic Fatty Liver Disease: A Single-Center Clinical Study

**DOI:** 10.1155/2018/8040262

**Published:** 2018-01-21

**Authors:** Ou Cai, Zhenpeng Huang, Ming Li, Chaoqun Zhang, Fengbo Xi, Shiyun Tan

**Affiliations:** ^1^Department of Gastroenterology, Renmin Hospital of Wuhan University, Wuhan, Hubei, China; ^2^Teaching and Research Section of Internal Medicine, College of Clinical Medicine, Xi'an Medical University, Xi'an, Shannxi, China; ^3^Enshi Prefecture Center for Disease Control and Prevention, Enshi, China

## Abstract

**Objective:**

To investigate the association between *Helicobacter pylori* (*H. pylori*) infection and nonalcoholic fatty liver disease (NAFLD).

**Methods:**

Data from 2051 participants who underwent ^13^C urea breath test and abdominal ultrasound examinations was collected. Participants were allocated to NAFLD risk group and NAFLD nonrisk group based on definite risk factors for NAFLD. The relationship between *H. pylori* infection and NAFLD was analyzed.

**Results:**

No significant difference was found between rates of *H. pylori* infection and NAFLD using the chi-square test (*P* = 0.30) or regression analysis (*P* = 0.70). There was no significant difference between rates of *H. pylori* infection with and without NAFLD (*P* = 0.47) in the NAFLD risk group or in the NAFLD nonrisk group (*P* = 0.59). There was no significant difference between rates of *H. pylori* infection in men (*P* = 0.69) and in women (*P* = 0.27) or in participants aged 18–40 years (*P* = 0.43), 41–65 years (*P* = 0.14), and ≥66 years (*P* = 0.66) with and without NAFLD in the NAFLD risk group or between the same sex or age groups (*P* = 0.82, *P* = 0.66, *P* = 0.24, *P* = 0.53, and *P* = 1.00, resp.) in the NAFLD nonrisk group.

**Conclusions:**

*H. pylori* infection does not appear to increase the NAFLD prevalence rate or to be associated with, or a risk factor for, NAFLD.

## 1. Introduction

Nonalcoholic fatty liver disease (NAFLD) is a type of liver injury induced by metabolic stress and is related to insulin resistance (IR) and hereditary susceptibility [1]. It is generally deemed to be the hepatic manifestation of metabolic syndrome [2]. NAFLD comprises simple nonalcoholic fatty liver (NAFL), nonalcoholic steatohepatitis (NASH) and NASH-associated liver cirrhosis, and hepatocellular carcinoma [1]. The incidence of and morbidity from NAFLD have increased rapidly worldwide, with corresponding increases in clinical and economic burden [3]. Apart from known and common risk factors, such as obesity, type 2 diabetes, hypertension, and dyslipidemia, it has recently been postulated that *Helicobacter pylori* infection is involved in the pathogenesis of insulin resistance (IR) [1, 4] and may be associated with NAFLD.

*H. pylori* is a Gram-negative bacterium which selectively colonizes the gastric mucosa [5] and is considered to be the main pathogenic bacteria involved in peptic ulcers, chronic active gastritis, mucosa-associated lymphoid tissue lymphoma, and gastric cancer [6–8]. Recently, *H. pylori* infection has been implicated in various nongastrointestinal diseases including idiopathic thrombocytopenic purpura, cardiovascular disease, type II diabetes, and iron deficiency anemia [9–11]. However, the findings of recent studies focusing on the relationship between *H. pylori* infection and NAFLD are inconsistent [12–15]. Our study used data from volunteers who underwent physical examinations in the Renmin Hospital of Wuhan University from June to December 2016 in order to analyze the association between *H. pylori* infection and NAFLD prevalence rates, to elucidate this relationship, and to provide a new strategy for treatment of NAFLD.

## 2. Materials and Methods

### 2.1. Study Subjects

**Figure 1:**
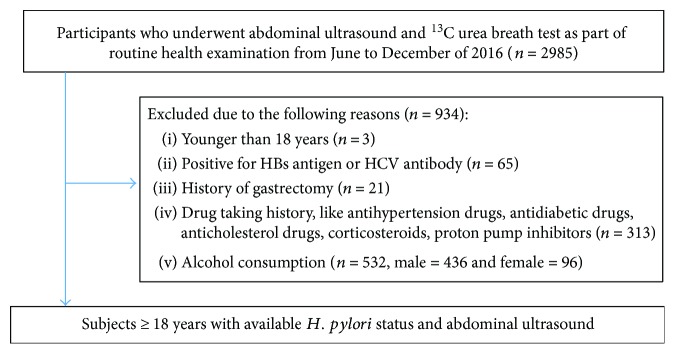
Of the 2985 participants attended, 2051 participants were analyzed in our study.

It was a cross-sectional study of Chinese asymptomatic adults who underwent physical examination in the Renmin Hospital of Wuhan University from June 2016 to December 2016 and entered into our study voluntarily (Figure 1). This study gained approval by the ethics committees of the Renmin Hospital of Wuhan University. We included participants who underwent ^13^C urea breath test and abdominal ultrasound (*n* = 2985). We excluded 934 subjects due to the following reasons: younger than 18 years old (*n* = 3); positive for HBs antigen or HCV antibody (*n* = 65); history of gastrectomy (*n* = 21); drug taking history, such as antihypertension drugs, antidiabetic drugs, anticholesterol drugs, corticosteroids, and proton pump inhibitors (*n* = 313); and alcohol consumption more than 140 g/week for male and 70 g/week for female (*n* = 532, male = 436 and female = 96). 2051 participants older than 18 years with available *H. pylori* status and abdominal ultrasound were finally analyzed. All participants were divided into NAFLD risk group and NAFLD nonrisk group according to definite NAFLD risk factors, including dyslipidemia (TG ≥ 1.70 mmol/L, TC ≥ 5.20 mmol/L, HDL-C < 1.00 mmol/L, and LDL-C ≥ 3.10 mmol/L), high blood glucose (FPG ≥ 6.10 mmol/L), high blood pressure (SBP ≥ 140 mmHg and DBP ≥ 90 mmHg), and BMI ≥ 24.90 kg/m^2^. Participants with any one or more of the eight items are included in the NAFLD risk group, and those with none of the eight are included in the NAFLD nonrisk group (Table 1).

### 2.2. General Information

We used uniformly trained nurses to enquire and fill in the uniformly designed epidemiologic questionnaires. In the morning, we routinely examined the height, weight, and blood pressure of every participant. The body mass index (BMI) was the weight (kg) divided by the square of height in meters.

### 2.3. Serum Biochemical Examination

Morning fasting venous blood of all participants was obtained to detect the levels of serum triglyceride (TG), cholesterol (TC), high-density lipoprotein cholesterol (HDL-C), low-density lipoprotein cholesterol (LDL-C), and fasting plasma glucose (FPG) with Backman AU5800 biochemical analyzer (Backman Coulter Commercial Enterprise (China) Co. Ltd., Beijing, China).

### 2.4. *H. pylori* Infection Test

The urea (^13^C) capsule breath test box (Shenzhen Zhonghe Headway Bio-Sci & Tech Co. Ltd., Shenzhen, Guangdong, China, Batch number 0520150904) and HUBT-20A breath test tester (Shenzhen Zhonghe Headway Bio-Sci & Tech Co. Ltd., Shenzhen, Guangdong, China) were used for detection. The procedure was as follows: (i) fill in the information of the participant in the two prepared collection bags; (ii) collect the air exhaled by the participant in quiet condition in one collection bag as the 0 minute sample; (iii) collect the air again after taking the urea (^13^C) capsule with 80 to 100 milliliter water for 30 minutes as the 30 minutes sample; (iv) detect the sample in 0 and 30 minutes in the HUBT-20A breath test tester to get the corresponding value; (v) use <delta>‰ to express the result and detection value equals <delta>‰ (30 minutes) minus <delta>‰ (0 minutes). When detection value was no less than 4.0, we regarded it as positive and according to the Fourth Chinese National Consensus Report on the management of *Helicobacter pylori* infection [16] (Table 1), the participant was in current infection.

### 2.5. Ultrasonic Measurement

Philips iE Elite and iE 33 (Philips China Investment Co., Beijing, China) were used for abdominal ultrasound by professional ultrasound doctors. According to the guidelines for the diagnosis and management of nonalcoholic fatty liver disease: update 2010 [1], participants who possessed two of the following three characteristics could be diagnosed as fatty liver: (i) the near-field echo of the liver is diffusely increased and more than the kidney; (ii) the intrahepatic duct structure is not clear; (iii) the far-field echo of the liver is decreased gradually (Table 1). Three professional ultrasound doctors were uniformly trained for the diagnosis of fatty liver, and each participant was measured by two of them. The diagnosis could not be made until at least two of the doctors made an agreement.

### 2.6. Statistical Analysis

Software SPSS version 18.0 was used for statistical analysis. Continuous data accorded with normal distribution were presented as average ± standard deviation, and categorical data were presented as number (percentage). Continuous variables were compared by *t*-test, and categorical variables were compared by chi-square test. Regression analysis was used to identify independent risk factors. *P* < 0.05 was regarded as indicating statistical significance.

## 3. Results

### 3.1. Baseline Characteristics

Baseline characteristics of the participants are shown in Table 2. The mean age was 38.11 ± 10.49 years, and the prevalence of NAFLD was 21.11% (433/2051). Compared to participants without NAFLD, those with NAFLD were more likely to be older and male, with higher BMI, SBP, and DBP, higher levels of TG, TC, LDL-C, and FPG, and lower levels of HDL-C (*P* = 0.00). However, there was no association between *H. pylori* infection and NAFLD (*P* = 0.30) (Table 2).

### 3.2. Risk Factors for NAFLD by Regression Analysis

Regression analysis was used to analyze the independent risk factors for NAFLD, and all the factors which were significant according to *t*-tests or chi-square tests were included, plus *H. pylori* infection. Analysis showed that sex (*P* = 0.01) and BMI and levels of TG, HDL-C, and FPG were independent risk factors for NAFLD (*P* = 0.00) but *H. pylori* infection was not (*P* = 0.70) (Table 3).

### 3.3. Association between *H. pylori* Infection and NAFLD

In the NAFLD risk group, the *H. pylori* infection rate among participants with NAFLD (34.16%) was slightly higher than that among participants without NAFLD (32.04%), although the difference was not significant (*P* = 0.47). There was also no significant difference between the rates of *H. pylori* infection and NAFLD in men (*P* = 0.69) and women (*P* = 0.27). Moreover, after age stratification, no significant difference was found between the rates of *H. pylori* infection and NAFLD in younger (18–40 years) (*P* = 0.43), middle-aged (41–65 years) (*P* = 0.14), and older (≥66 years) participants (*P* = 0.66). In the NAFLD nonrisk group, the *H. pylori* infection rate among participants with NAFLD (25.00%) was lower than that among participants without NAFLD (30.10%), although the results were not significant (*P* = 0.59). Similar results were also obtained for men (*P* = 0.82), women (*P* = 0.60), younger (*P* = 0.24), middle-aged (*P* = 0.53), and older participants (*P* = 1.000) (Table 4).

## 4. Discussion

NAFLD is a common metabolic disorder that affects approximately 13.48%–31.79% of the general population [3], although its mechanism remains unclear. Genetic, environmental, and metabolic factors may be involved in the pathogenesis of NAFLD, and some recent research provides insights into the link between *H. pylori* infection and NAFLD. In 2008, Cindoruk et al. [17] found *H. pylori* 16S rDNA in a biopsy taken from a 44-year-old woman with NASH and, in 2009, Pirouz et al. [18] found 5 H*. pylori* 16S rRNA-positive patients among 11 biopsy-proven NAFLD patients, compared with 2 positive patients among 13 controls. This indicated that *H. pylori* could infect the liver and aroused academic interest. However, it has been difficult to culture *H. pylori* from the liver [19], suggesting that it cannot successfully colonize the liver and is therefore unlikely to cause damage.

Research into the mechanism underlying the relationship between *H. pylori* infection and NAFLD has mainly focused on IR. In 2011, a systematic review conducted by Polyzos et al. [4] concluded that there was a positive association between *H. pylori* infection and IR but significant heterogeneity had been found between studies and further research was therefore needed. In 2013, Abenavoli et al. [20] conducted a study that found that serum insulin levels and homeostatic model assessment- (HOMA-) IR were lower after *H. pylori* eradication and concluded that *H. pylori* eradication might improve IR. However, the intrinsic mechanism still remained obscure. In 2013, Polyzos et al. [12] first showed that liver biopsy-proven NAFLD patients had significantly higher anti-*H. pylori* IgG levels and demonstrated that *H. pylori* infection might contribute to NAFLD directly or indirectly via IR, since NAFLD is an independent predictor of IR. However, this study included a relatively small number of participants (28), and *H. pylori* seropositivity, which cannot distinguish between current and past infection, was used to detect *H. pylori* infections. All these studies indicate that there is a positive association between *H. pylori* infection and IR but have not identified a definite pathophysiological mechanism.

No association between *H. pylori* infection and NAFLD was found in two recent large clinical trials. Okushin et al. [13] conducted a large-scale study in Japan in 2015 of 13,737 participants. The authors concluded that BMI, platelet count, and serum alanine aminotransferase levels were associated with NAFLD but *H. pylori* infection was not. In 2016, Baeg et al. [14] analyzed data from 3663 patients and concluded that smoking and C-reactive protein concentration were risk factors for NAFLD but *H. pylori* infection was not. Our study also found that *H. pylori* infection was not associated with NAFLD, even after stratifying by sex and age.

Our study has some limitations. The first is that as a single-center cross-sectional study, it was difficult to infer causation and our study therefore represents a low level of evidence. The second is the use of abdominal ultrasound to diagnose NAFLD, since the results are operator dependent. However, we attempted to mitigate error by ensuring that the doctors in our study were trained according to the same protocol and a confirmed diagnosis was based on the opinion of at least two doctors. A third limitation is that we did not grade the severity of NAFLD or distinguish NASH from NAFL. Sumida et al. [15] found that the prevalence of NASH and the grade of hepatocyte ballooning were higher in *H. pylori*-seropositive patients, indicating that *H. pylori* infection may act as a contributing factor in the progression of NAFL to NASH but not in early-stage NAFLD. In spite of its limitations, our study has some advantages. To our knowledge, ours is the first study to group data by definite risk factors for NAFLD into an NAFLD risk group and NAFLD nonrisk group. We also excluded the effect of age and sex by analyzing and stratifying the data and found a similar trend of no association between the stratified data; this strengthens our conclusions.

To conclude, although, to date, the association between *H. pylori* infection and NAFLD has been controversial and our research found no association despite limitations in the design of our study. Our large cross-sectional study focused on the relationship between *H. pylori* infection and NAFLD. We found that *H. pylori* infection did not associate with NAFLD, even when the data were stratified according to sex and age. Our findings therefore indicate that *H. pylori* infection has no association with NAFLD. Further large-scale multicenter prospective studies are urgently needed to investigate whether there is any association between *H. pylori* infection and NAFLD and to clarify the intrinsic mechanisms involved.

## Figures and Tables

**Table 1:** Definition of *H. pylori* infection, NAFLD, and NAFLD risk group.

	Item	Value	Standard
*H. pylori* infection	^13^C urea breath test	<delta>‰ (30 minutes) minus <delta>‰ (0 minutes)	Positive: ≥4 Negative: <4

NAFLD	Abdominal ultrasound	(i) The near-field echo of the liver is diffusely increased and more than the kidney. (ii) The intrahepatic duct structure is not clear. (iii) The far-field echo of the liver is decreased gradually.	Positive: any two of the three satisfied Negative: none or only one satisfied

NAFLD risk or not	Blood lipid	TG ≥ 1.70 mmol/L	Risk group: one or more of the eight satisfied Nonrisk group: none of the eight satisfied
		TC ≥ 5.20 mmol/L	
		HDL-C < 1.00 mmol/L	
		LDL-C ≥ 3.10 mmol/L	
	Fast plasma glucose	FPG ≥ 6.10 mmol/L	
	Blood pressure	SBP ≥ 140 mmHg	
		DBP ≥ 90 mmHg	
	Body mass index	BMI ≥ 24.90 kg/m^2^	

NAFLD: nonalcoholic fatty liver disease; TG: triglyceride; TC: total cholesterol; HDL-C: high-density lipoprotein cholesterol; LDL-C: low-density lipoprotein cholesterol; FPG: fast plasma glucose; SBP: systolic blood pressure; DBP: diastolic blood pressure; BMI: body mass index.

**Table 2:** Baseline characteristics of study participants.

	All (*n* = 2051)	NAFLD (−) (*n* = 1618)	NAFLD (+) (*n* = 433)	Statistics	*P* value
Age (y)	38.11 ± 10.49	37.25 ± 10.38	41.31 ± 10.30	*t* = −7.24	0.00
Sex (male/female)	714/1337	446/1172	268/165	*χ* ^2^ = 177.39	0.00
BMI (kg/m^2^)	23.45 ± 3.05	22.63 ± 2.55	26.52 ± 2.77	*t* = −27.66	0.00
SBP (mmHg)	118.30 ± 14.52	116.37 ± 13.71	125.53 ± 15.18	*t* = −12.07	0.00
DBP (mmHg)	71.11 ± 10.07	69.77 ± 9.62	76.11 ± 10.18	*t* = −12.03	0.00
TG (mmol/L)	1.29 ± 1.15	1.06 ± 0.66	2.18 ± 1.91	*t* = −12.01	0.00
TC (mmol/L)	4.43 ± 0.80	4.35 ± 0.77	4.73 ± 0.85	*t* = −8.46	0.00
HDL-C (mmol/L)	1.34 ± 0.36	1.40 ± 0.36	1.11 ± 0.24	*t* = 19.29	0.00
LDL-C (mmol/L)	2.44 ± 0.67	2.37 ± 0.64	2.73 ± 0.70	*t* = −10.14	0.00
FPG (mmol/L)	5.19 ± 0.98	5.07 ± 0.56	5.66 ± 1.76	*t* = −6.96	0.00
*H. pylori* infection (−/+)	1406/645	1118/500	288/145	*χ* ^2^ = 1.06	0.30

Values are expressed as means ± standard deviation. NAFLD: nonalcoholic fatty liver disease; BMI: body mass index; SBP: systolic blood pressure; DBP: diastolic blood pressure; TG: triglyceride; TC: total cholesterol; HDL-C: high-density lipoprotein cholesterol; LDL-C: low-density lipoprotein cholesterol; FPG: fast plasma glucose.

**Table 3:** Independent risk factors for NAFLD by regression analysis.

	*B*	SE	Wald	Exp (*B*)	95% CI	*P*
Sex	−0.40	0.15	6.83	0.69	0.49–0.90	0.01
BMI	0.39	0.03	163.17	1.47	1.39–1.56	0.00
TG	0.55	0.11	26.31	1.73	1.40–2.14	0.00
HDL-C	−1.32	0.38	12.19	0.27	0.13–0.56	0.00
FPG	0.42	0.10	16.14	1.52	1.24–1.86	0.00
*H. pylori* infection	−0.06	0.15	0.15	0.94	0.70–1.27	0.70

BMI: body mass index; TG: triglyceride; HDL-C: high-density lipoprotein cholesterol; FPG: fast plasma glucose.

**Table 4:** Association analysis between *H. pylori* infection and NAFLD.

	NAFLD risk group (*n* = 1072)			NAFLD nonrisk group (*n* = 979)		
	Positive	Negative	*χ* ^2^ (*P*)	Positive	Negative	*χ* ^2^ (*P*)
*Whole participate*			0.51 (0.47)			0.38 (0.59)
*H. pylori* (+)	137 (38.92%)	215 (61.18%)		8 (2.73%)	285 (97.27%)	
*H. pylori* (−)	264 (36.67%)	456 (63.33%)		24 (3.50%)	662 (96.50%)	
*In different sex*						
Male			0.16 (0.69)			0.05 (0.82)
*H. pylori* (+)	81 (47.65%)	89 (52.35%)		3 (5.36%)	53 (94.64%)	
*H. pylori* (−)	176 (45.83%)	208 (54.17%)		8 (7.69%)	96 (92.31%)	
Female			1.23 (0.27)			0.28 (0.60)
*H. pylori* (+)	56 (30.77%)	126 (69.23%)		5 (2.11%)	232 (97.89%)	
*H. pylori* (−)	88 (26.19%)	248 (73.81%)		16 (2.75%)	566 (97.25%)	
*In different age*						
18–40			0.62 (0.43)			1.36 (0.24)
*H. pylori* (+)	54 (32.53%)	112 (67.47%)		3 (1.50%)	197 (98.50%)	
*H. pylori* (−)	138 (36.03%)	245 (63.97%)		18 (3.48%)	499 (96.52%)	
41–65			2.16 (0.14)			0.40 (0.53)
*H. pylori* (+)	79 (44.63%)	98 (55.37%)		5 (5.43%)	87 (94.57%)	
*H. pylori* (−)	122 (37.89%)	200 (62.11%)		5 (3.01%)	161 (96.99%)	
≥66			0.20 (0.66)			— (1.00)
*H. pylori* (+)	4 (44.44%)	5 (55.56%)		0 (0)	1 (100%)	
*H. pylori* (−)	4 (26.67%)	11 (73.33%)		1 (33.33%)	2 (66.67%)	
